# Effect of Multi-Variant Thermal Treatment on Microstructure Evolution and Mechanical Properties of AlSi10Mg Processed by Direct Metal Laser Sintering and Casting

**DOI:** 10.3390/ma15030974

**Published:** 2022-01-27

**Authors:** Krzysztof Żaba, Lechosław Tuz, Piotr Noga, Stanislav Rusz, Rostislav Zabystrzan

**Affiliations:** 1 Department of Metal Working and Physical Metallurgy of Non-Ferrous Metals, Faculty of Non-Ferrous Metals, AGH University of Science and Technology, Al. Mickiewicza 30, 30-059 Kraków, Poland; 2 Department of Physical & Powder Metallurgy, Faculty of Metal Engineering and Industrial Computer Science, AGH University of Science and Technology, Al. Mickiewicza 30, 30-059 Kraków, Poland; ltuz@agh.edu.pl; 3 Department of Materials Science and Engineering of Non-Ferrous Metals, Faculty of Non-Ferrous Metals, AGH University of Science and Technology, Al. Mickiewicza 30, 30-059 Kraków, Poland; pionoga@agh.edu.pl; 4 Department of Mechanical Technology, Faculty of Mechanical Engineering, VŠB—Technical University of Ostrava, 17 Listopadu 15, 708-33 Ostrava-Poruba, Czech Republic; stanislav.rusz@vsb.cz; 5 SteelTec CZ, s. r. o., Ul. Prumyslova 700, 739-55 Trinec, Czech Republic; rostislav.zabystrzan@steeltec.cz

**Keywords:** AlSi10Mg alloys, direct metal laser sintering, casting, heat treatment, microstructure, mechanical properties, computer tomography, porosity

## Abstract

This article presents a study on the influence of temperature and time of multi-variant heat treatment on the structure and properties of materials produced in direct metal laser sintering (DMLS) and casting technology. The materials were manufactured in the form of cuboidal elements with a cross-section of 1.5 mm × 15 mm and a length of 60 mm. The samples prepared in this way had a similar volume, but due to the production technology the metal crystallization took place at different rates and directions. In the cast, the direction of heat transfer was toward the mold, and the DMLS was directed locally layer by layer. The small thickness of the cast material allowed reaching conditions similar to the DMLS cooling process. Both DMLS and cast samples show similar mechanical properties (hardness) achieved after long ageing time, i.e., 16 h at 170 °C. The maximum hardness was observed for 8 h. In the DMLS samples, in contrast to cast samples, no lamellar precipitates of silicon were observed, which indicates their better resistance to cracking

## 1. Introduction

Due to the favorable ratio of mechanical properties to density, easy machining and high fatigue strength, aluminum alloys are used in the manufacturing of many structural elements in the form of castings, or for forging or welding. Aluminum alloys are also important materials in the automotive and aviation industries. Among the many grades of Al alloys, a group of casting alloys with excellent castability, high strength, ductility and good weldability can be distinguished (i.e., Al-Si, AlSi5Cu2 and AlSi10Mg) [1]. The addition of Si lowers the melting point of the alloys and provides high castability, which allows manufacturing elements with very complex shapes and thin walls and can reduce defects such as cracking, shrinkage and porosity [2]. The addition of Mg increases the strength and corrosion resistance of the alloys [2].

Cast aluminum alloys are used for the production of engine hulls and heads as well as rotors of fans for the engine coolers. As is known, the casting method used for the production of these products, despite its undeniable advantages, also has a number of disadvantages related to the properties of the materials, their structure and production costs. For this reason, in the last few years, a high increase in the interest of both scientists and the industrial community in additive manufacturing technology (AM) has been noticed. The AM methods include metal powder bed fusion 3D printing (SLS, SLM, DMP, DMLS) [3], directed energy deposition (DED) [4], metal filament extrusion (FFF, FDM) [5], material jetting and binder jetting [6]. SLM is widely accepted by the industry for its possible usage in the production of complex metal components in aviation, the automotive industry, defense and biomedical applications [7].

Comparing the casting and SLM processes, it needs to be stated that there are fundamental differences between the microstructures of the materials obtained due to the layered thermal cycle, focused input energy and rapid cooling during the production of layers in the SLM process. According to the authors of [8], the maximum cooling rate of the SLM process is 3–4 orders of magnitude higher than that achieved during conventional casting processes. Differences in microstructure affect the properties of the material and ultimately affect the properties of the final product. The mostly tested alloys produced by SLM are AlSi10Mg, AlSi12, A356 and A357. Recently, in many studies, the effects of process parameter optimization [9,10,11], heat treatment [10,12,13,14,15,16,17,18,19,20,21,22], build orientation [12,17,20,23,24,25,26,27,28,29,30,31] and numerical studies [32,33] were investigated. The most frequently tested material is AlSi10Mg alloy [34,35]. Among the studies carried out on samples of this material obtained in the SLM process, the selected properties are presented, i.e., tensile strength [10,12,13,19,22,23,28,29,30,31,36,37,38], microhardness [16,17,19,22,33,38], nanohardness [13,15,23,31,39], compressive strain [11,13,23] fatigue strength [14,26,31,40], wear resistance [16,41] and fracture or toughness [24,26,29,32,41].

Heat treatment is one of the basic processes to achieve the desired property, such as the ductility of parts produced in SLM technology. Li et al. [21] examined AlSi10Mg samples made by the SLM method and tested them at −70 °C for mechanical properties. The fish-scale structure morphology along the building direction and oval structures on the vertical side of the building direction were observed. Takata et al. [42] investigated the microstructure and mechanical properties of AlSi10Mg samples produced by the SLM method, heat treated at a temperature above 300 °C (annealed). They found that at elevated temperatures fine Si particles would inhibit the migration of grain boundary. Moreover, the fine Si particles enhance the strain-hardening in the α-Al matrix, resulting in crack initiation. Moreover, they found that the tensile strength is isotropic, while the ductility is anisotropic, with the anisotropic properties declining after heat treatment at 530 °C. Girelli et al. [43] investigated the effect of temperature, solution treatment and ageing on the microstructure, microhardness and density of AlSi10Mg samples prepared by the SLM method. They also tested AlSi10Mg samples produced by gravity casting under the same heat treatment conditions. It was found that the SLM samples had a fine-grained microstructure and showed better mechanical properties than the gravity-cast samples due to the refinement of Si grains and nanoparticles. Despite many studies on the influence of heat treatment on the structure and properties of AlSi10Mg obtained by SLM, there are no reports on the influence of the time of ageing on the mechanical properties and structure as a result of prolonged exposure to elevated temperature.

This paper presents the influence of temperature and time of multi-variant heat treatment on the structure and properties of materials produced in direct metal laser sintering (DMLS) and casting technology. The materials were manufactured in the form of cuboidal elements with a cross-section of 1.5 mm × 15 mm and a length of 60 mm. The samples prepared in this way had a similar volume, but due to the production technology the metal crystallization took place at different rates and directions. In the cast, the direction of heat transfer was toward the mold and the DMLS was directed locally layer by layer. The small thickness of the cast material allowed reaching the conditions similar to the DMLS cooling process.

## 2. Materials and Methods

**Figure 1:**
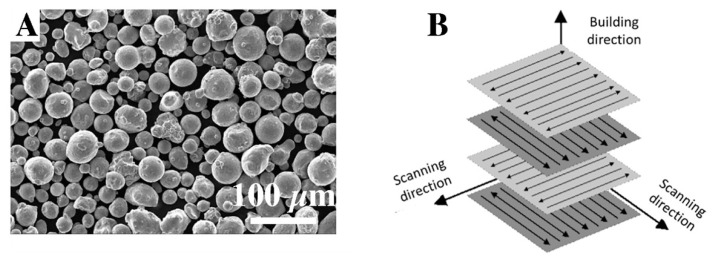
AlSi10Mg powder (**A**), scanning strategy of DMLS process (**B**).

The casting process was carried out by melting and cooling in the steel mould. The samples of AlSi10Mg alloys were obtained by casting technology at 720 °C using a resistance furnace. In AM technology by DMLS method the EOS device M290 was used. For testing, EOS Aluminum AlSi10Mg, provided by the EOS GmbH Electro Optical Systems in the form of a gas-atomized metal in homogeneous spherical shape powder, was used. The (range) mean particle size of the powder was (20 μm–90 μm) 36 μm. The SEM images of the powder are presented in Figure 1A. The scanning strategy is shown in Figure 1B. The scanning angle was 90° in relation to the previous layer. The DMLS process parameters are presented in Table 1.

**Figure 2:**
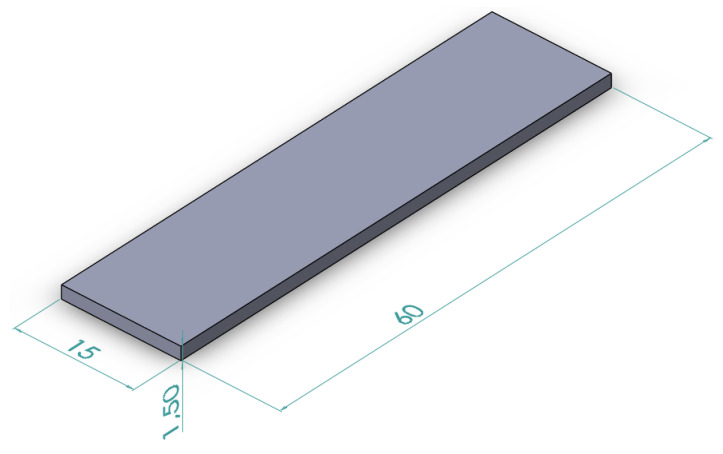
Scheme of AlSi10Mg sample.

**Figure 3:**
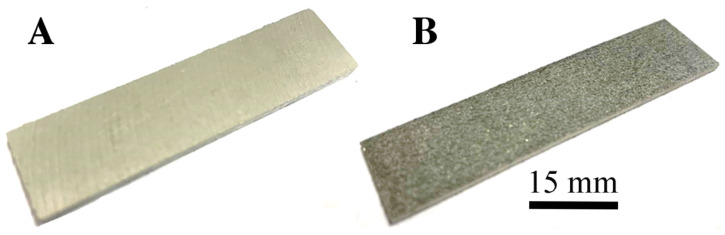
Cast (**A**) and DMLS (**B**) AlSi10Mg alloy samples.

The 3D model of the test samples is shown in Figure 2. Figure 3 shows an exemplary sample made by casting (Figure 3A) and by the DMLS method (Figure 3B).

The samples were made in the form of flat bars (cuboidal) with the dimensions of 1.5 mm × 15 mm × 60 mm (Figure 2 and Figure 3) and then electroerosively cut with the use of a WEDM cutting machine and a BP05d electro-erosion machine (Zakład Automatyki Przemysłowej B.P., Konskie, Poland) into smaller sections (5 mm × 5 mm × 1.5 mm). All of the samples prepared in this way were subjected to supersaturation at the temperature of 570 °C/2 h and ageing at the temperature of 170 °C. The ageing times were 2 h, 4 h, 6 h, 8 h and 16 h, respectively.

In order to reveal the accuracy of the sample production process and the mapping of the geometric model, they were subjected to 3D scanning with the use of a GOM ATOS Core 3D scanner. Due to the typical porosity of casting alloys, the samples were tested using computed tomography (CT) with the use of a Phoenix v|tomex m300 (GE dynamic 41|100 detector 410 mm × 410 mm (16″ × 16″), 100 µm pixel size, 4048 × 4048 pixels (16 MP) for doubled CT resolution). The porosity of samples was carried out with the use of myVGL 3.5 software (Volume Graphics GmBH, Heidelberg, Germany). The process of determining the volume of air voids was carried out on a reconstructed 3D solid. The VGDefX algorithm in voids mode was used. The analysis reveals air voids in the entire body or in its fragment indicated by use. The assessment is performed visually and on the basis of tables and graphs. Depending on the size of the void, the pores are shown with different colors on the cross-sections and in the 3D view. Additionally, it is decisive to present the pore distribution as a function of the appropriate coordinate. Based on the analysis of the radiographic image, a 3D model of the cast sample and DMLS was made with the distribution of porosity in the sample volume.

**Figure 4:**
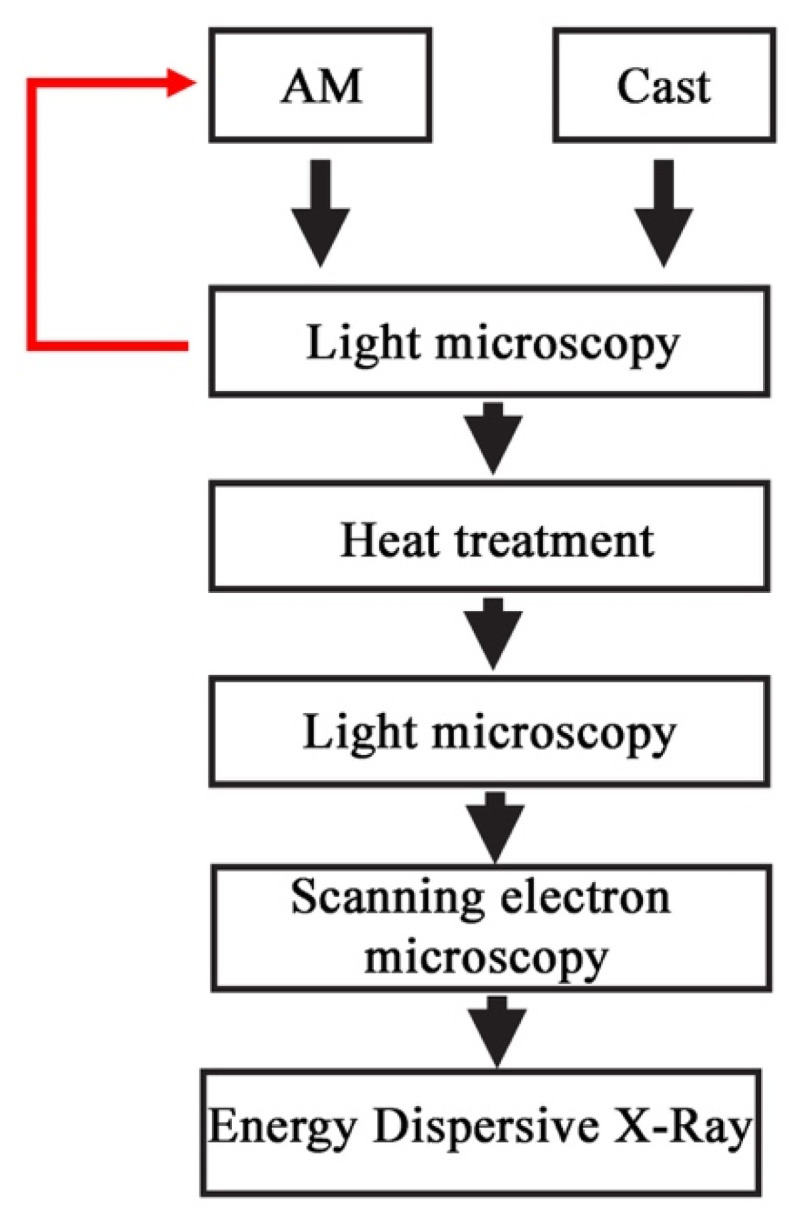
Microscopic examination schedule.

After the heat treatment processes, the metallographic sections were prepared. Samples were polished—first with 600–2000 grit abrasive papers and then in 0.04 mm gradation OPS slurry. Figure 4 shows the research schedule. In order to reveal the structure, microscopic examinations were carried out using light microscopy (LM) and scanning electron microscopy (SEM). For light microscopy examination, the Leica LM/DM (Leica, Wetzlar, Germany) light microscope was used and for SEM examination the Phenom XL (Thermo Fisher Scientific, Waltham, MA, USA) and Hitachi SU-70 (Hitachi Ltd., Tokyo, Japan) microscopes were used.

In order to reveal the distribution of elements and identify the basic phases, maps of the distribution of alloying elements were made. The tests were performed using the SEM microscope Hitachi SU-70 (Hitachi Ltd., Tokyo, Japan) and the EDAX detector.

## 3. Results

**Figure 5:**
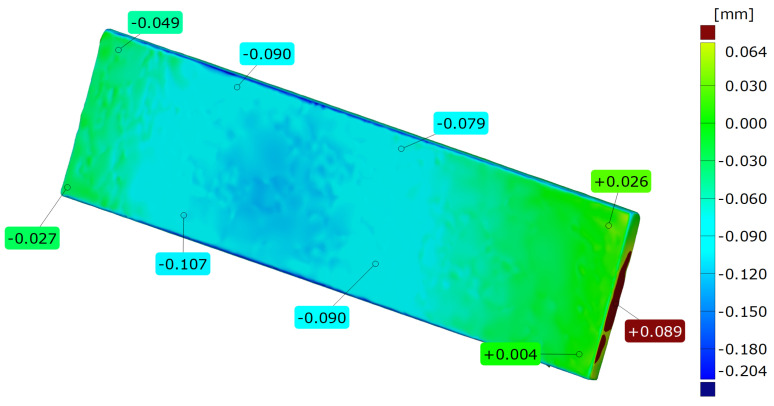
The results of 3D optical scanning tests of samples made of AlSi10Mg-cast.

**Figure 6:**
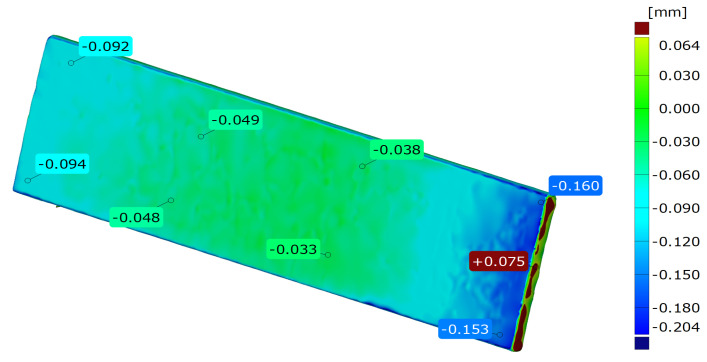
The results of 3D optical scanning tests of samples made of AlSi10Mg–DMLS.

The results of the 3D laser scanning tests of samples made of AlSi10Mg-cast (Figure 5) and DMLS (Figure 6). The geometric measurements of the samples show that they show high dimensional accuracy both in the condition after casting and when made by the DMLS method. The dimensions change due to shrinkage during the cooling of samples, where the maximum deviation of <0.1 mm for cast samples was achieved, and for DLMS it was <0.5 mm in the central part and less than 0.1 mm at the edges. The performed measurements indicate a very high dimensional accuracy of the samples made using the DMLS method.

**Figure 7:**
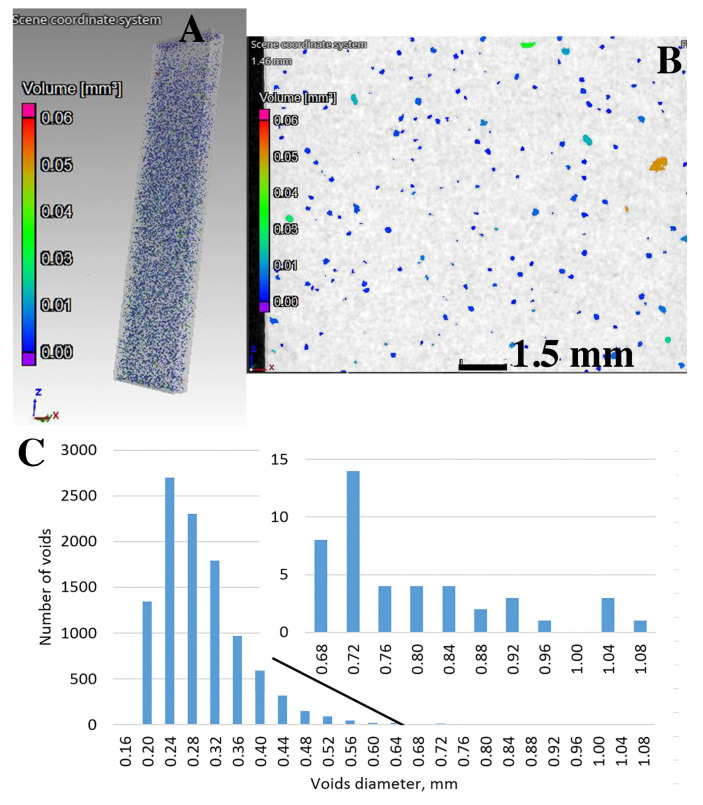
The results of CT tests of samples made of AlSi10Mg-cast: (**A**) sample 3D model, (**B**) porosity in cross-section and (**C**) diameter and volume distribution of separate pores.

**Figure 8:**
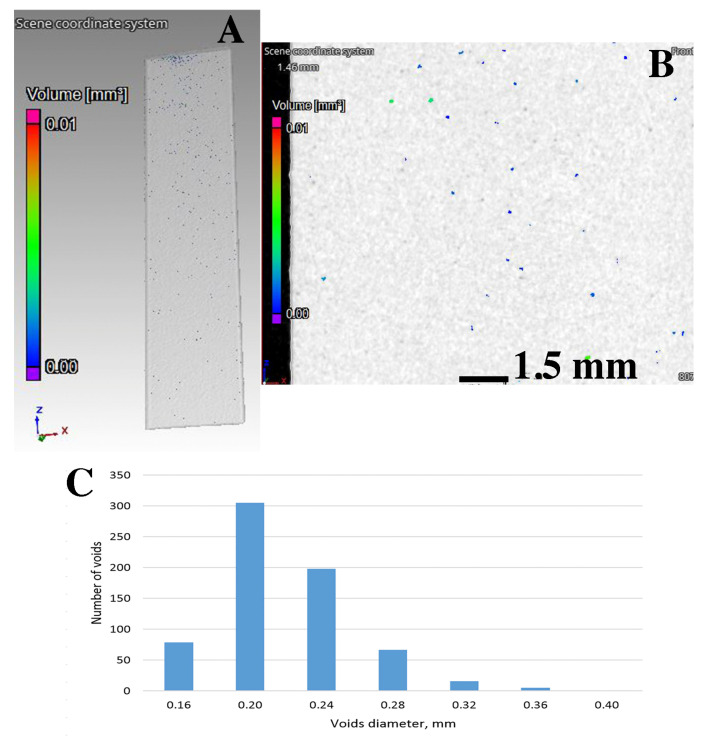
The results of CT tests of samples made of AlSi10Mg–AM: (**A**) sample 3D model, (**B**) porosity in cross-section and (**C**) diameter and volume distribution of separate pores.

The analysis of the porosity distribution model shows that the cast material is characterized by significant porosity. The voids are evenly distributed throughout the sample volume (Figure 7). The analysis of the volume distribution of individual voids shows that voids with a diameter below 0.5 mm and with a volume below 0.025 mm^3^ dominate (Figure 7C. Larger diameter voids represent only a small proportion of all those observed. The total proportion of porosity in the samples does not exceed 2.38%. For samples made with the DLMS method, the share of gas voids is significantly lower (Figure 8). Single voids of very small sizes (diameter < 0.25 mm, volume < 0.002 mm^3^) are observed (Figure 8C). The total porosity fraction obtained is less than 0.03%.

**Figure 9:**
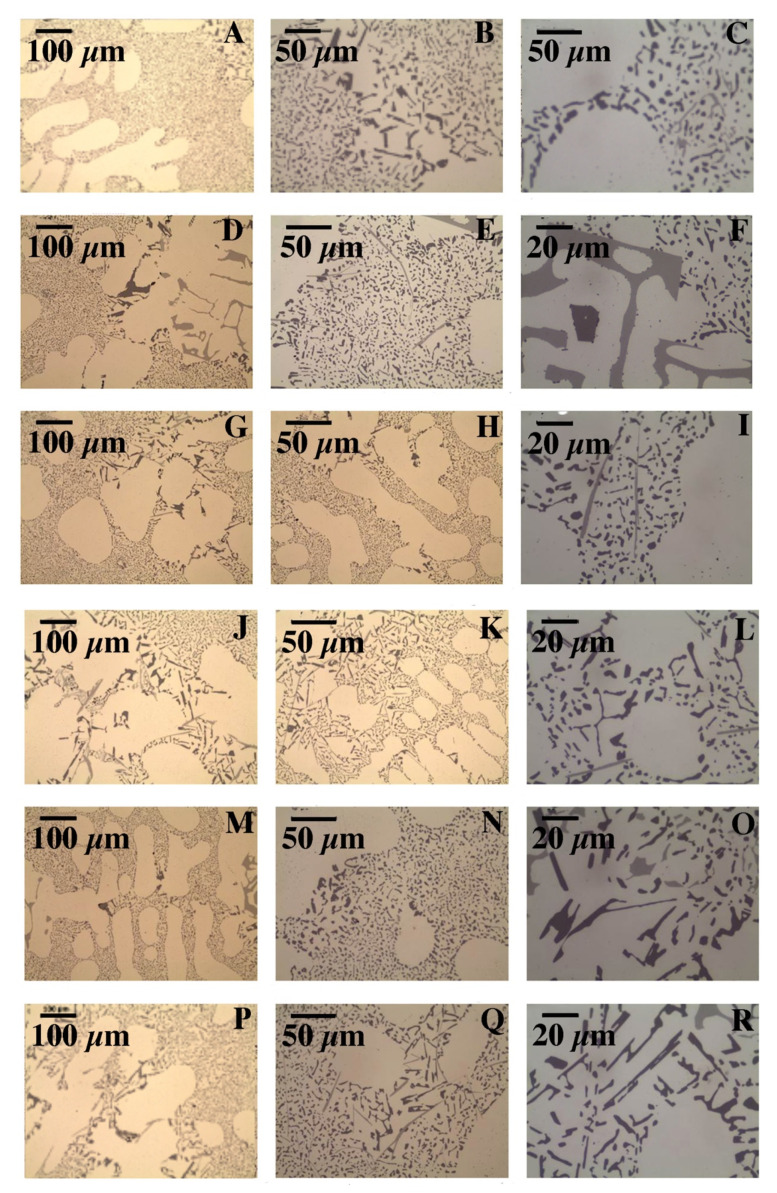
Microstructure of cast samples before and after heat treatment (LC): (**A**) after casting, (**B**,**C**) after supersaturation, (**D**–**F**) after supersaturation and ageing—2 h, (**G**–**I**) after supersaturation and ageing—4 h, (**J**–**L**) after supersaturation and ageing—6 h, (**M**–**O**) after supersaturation and ageing—8 h and (**P**–**R**) after supersaturation and ageing—16 h.

**Figure 10:**
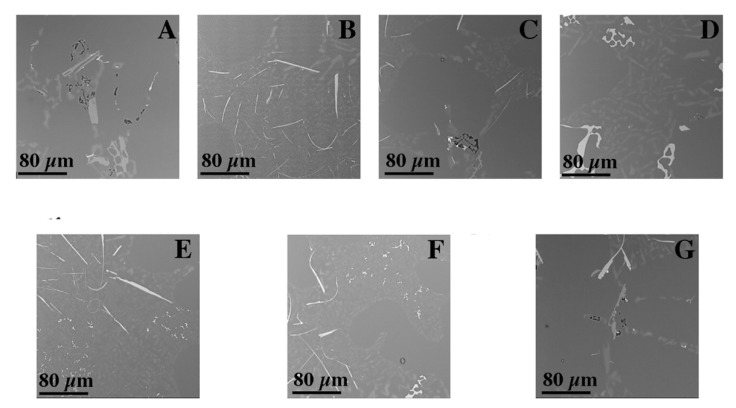
Microstructure of cast samples before and after heat treatment (SEM): (**A**) after casting, (**B**) after supersaturation, (**C**) after supersaturation and ageing—2 h, (**D**) after supersaturation and ageing—4 h, (**E**) after supersaturation and ageing—6 h, (**F**) after supersaturation and ageing—8 h and (**G**) after supersaturation and ageing—16 h.

**Figure 11:**
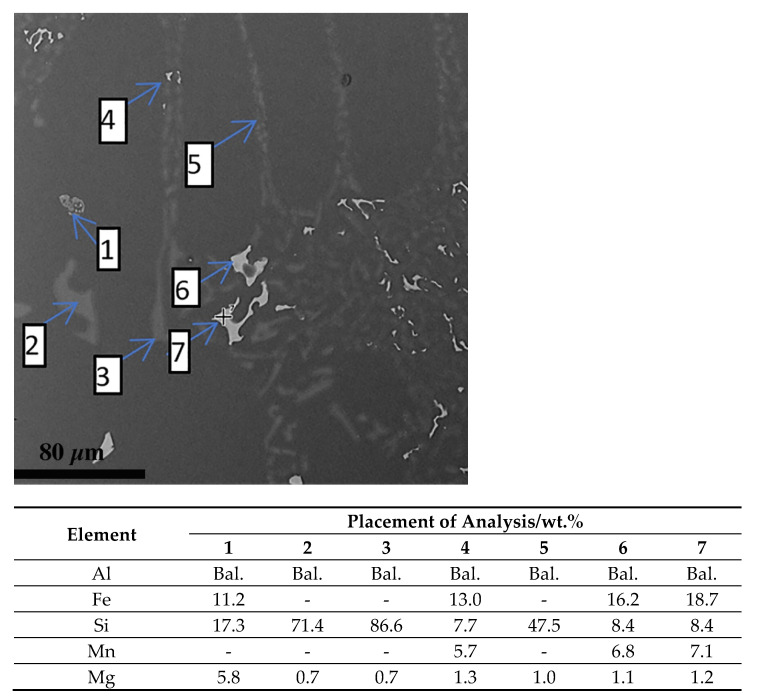
Chemical composition analysis of indicated phases after casting (SEM–EDS).

The microscopic tests performed show the dendritic structure of the samples after the casting process, typical for the crystallization of the castings. In the interdendritic regions, lamellar and globular precipitations were observed, indicating segregation of the alloying elements during crystallization and their pushing into the interdendritic regions (Figure 9 and Figure 10). Regardless of the thermal treatment performed, the dendrite cores remained free from precipitation, and the morphology of the precipitates did not change. Chemical composition analysis (EDS) shows that these precipitates, depending on the morphology, are rich in silicon, iron and magnesium (Figure 11).

**Figure 12:**
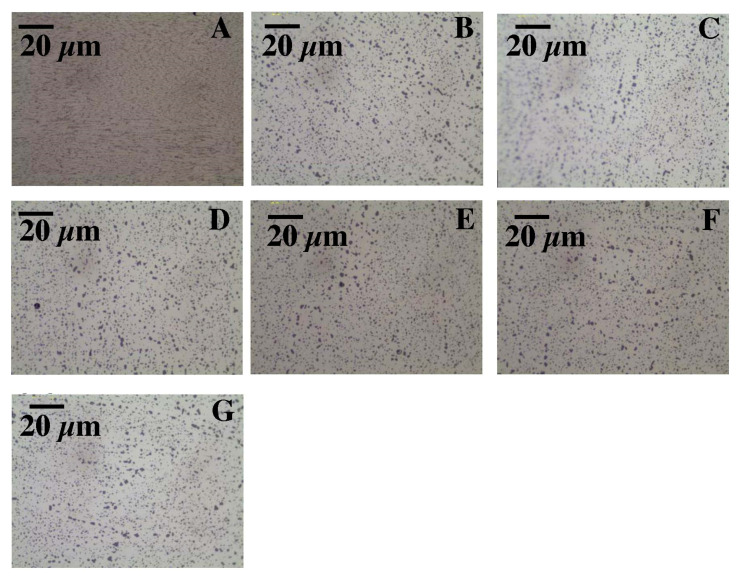
Microstructure of DMLS samples before and after heat treatment (LM): (**A**) after DMLS, (**B**) after supersaturation, (**C**) after supersaturation and ageing—2 h, (**D**) after supersaturation and ageing—4 h, (**E**) after supersaturation and ageing—6 h, (**F**) after supersaturation and ageing—8 h and (**G**) after supersaturation and ageing—16 h; Observation of the cross-section in the middle of thickness for each sample.

**Figure 13:**
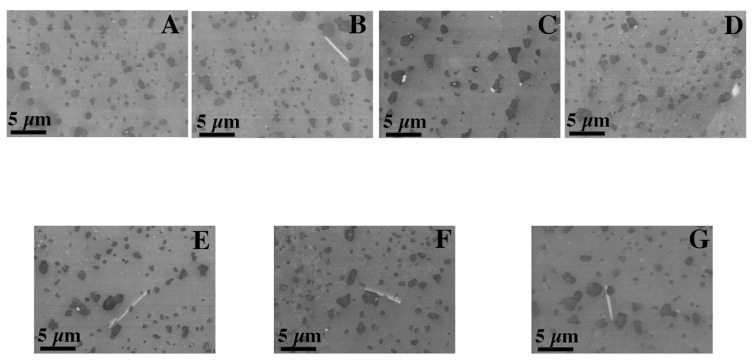
Microstructure of cast samples before and after heat treatment (SEM): (**A**) after DMLS, (**B**) after supersaturation, (**C**) after supersaturation and ageing—2 h, (**D**) after supersaturation and ageing—4 h, (**E**) after supersaturation and ageing—6 h, (**F**) after supersaturation and ageing—8 h and (**G**) after supersaturation and ageing—16 h.

**Figure 14:**
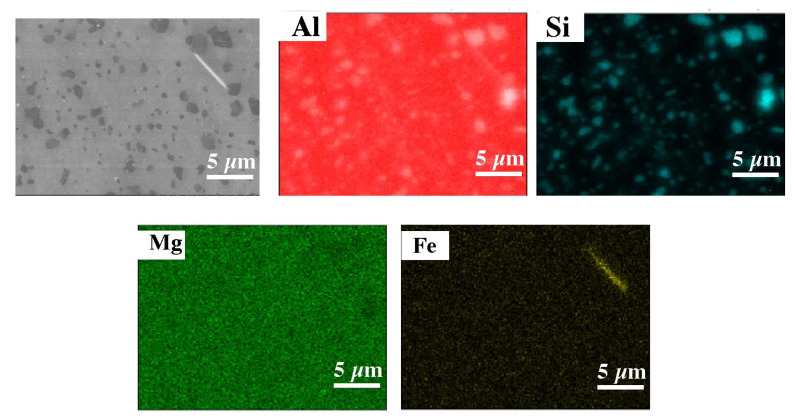
Map of the distribution of elements for the sample after supersaturation (EDS).

**Figure 15:**
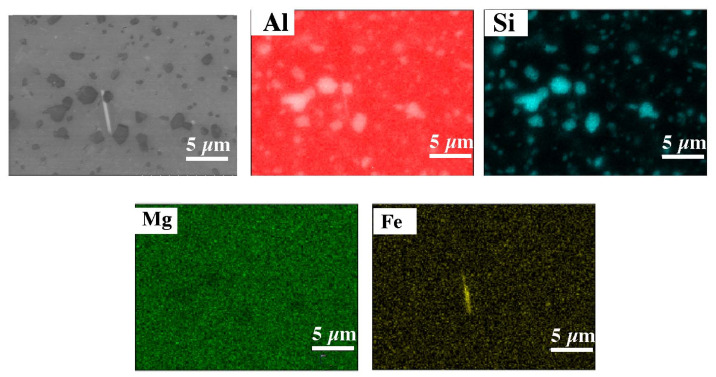
Map of the distribution of elements for the sample after supersaturation and 16 h ageing (EDS).

In the DMLS samples, the structure is a fine-crystalline structure with outlined areas of melted and crystallized powder. As a result of the process, the Si particles were significantly fragmented compared with the microstructure after casting. The diameter of the silicon-rich particles is about 1 um, additionally the morphology of these particles has changed, the particles have become globular, which compared with sharp Si edges in the cast material may have a costly impact on mechanical properties. The separations in the post-print state are evenly distributed. The process of supersaturation, as well as supersaturation and ageing, regardless of the process time, caused a slight increase in the size of the precipitates in relation to the stand after printing, and with a long ageing time (16 h) the grains were scratched (Figure 12 and Figure 13). The maps of the distribution of alloying elements for the samples in the state after supersaturation and ageing did not show any significant changes in the alloy (Figure 14 and Figure 15).

It was observed that Fe-reach precipitates are both needle-shaped and very fine. Different time of ageing did not influence the needle-shaped precipitation, but for fine, globular precipitations small differences were observed. For 4 h ageing, the globular precipitations increased.

**Figure 16:**
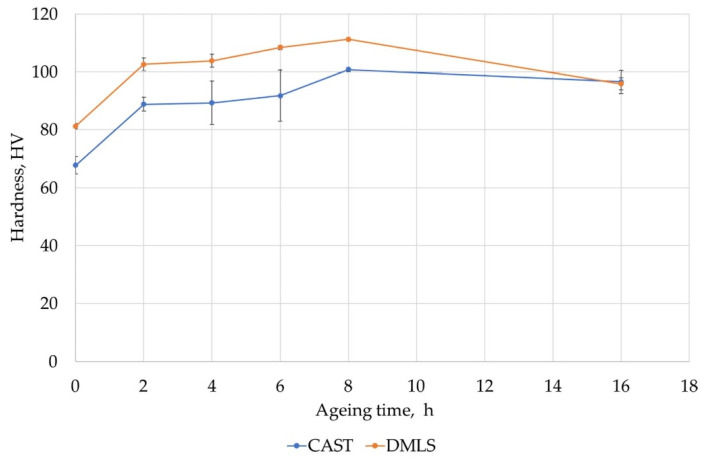
Hardness distribution for cast and DMLS samples depending on the ageing time (temperature 170 °C).

The hardness measurements performed for the cast samples showed an increase in hardness for the ageing times of 2, 4, 6 and 8 h, while for 16 h, the hardness was similar to 8 h of ageing (to 96 HV0.2). In the as-cast condition, the hardness was 65 HV0.2 and after supersaturation it was 75 HV0.2. The highest value was recorded for the ageing time of 8 h and it amounts to 100 HV0.2 (Figure 16).

The DMLS samples after supersaturation had a hardness of 80 HV0.2, which increased after ageing for 8 h to 113 HV0.2. After 16 h of ageing, a decrease in hardness to 98 HV0.2 was observed (Figure 16), which indicates the beginning of the overageing process. Further extending heat treatment time results in further hardness decrease.

**Figure 17:**
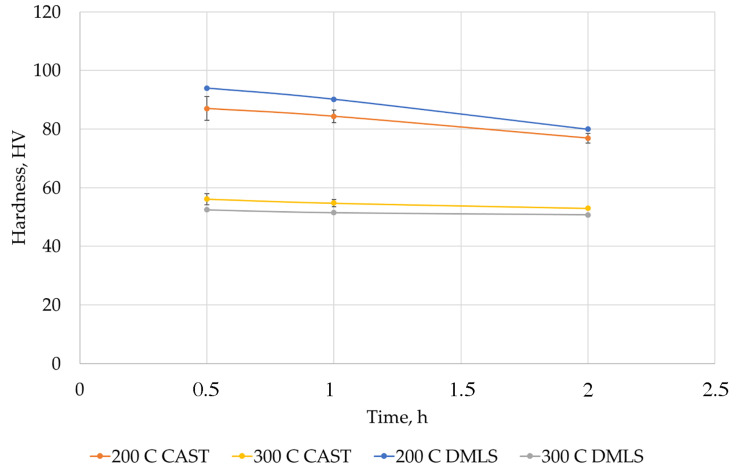
Hardness distribution for cast and printed samples depending on the ageing time and temperature.

The tests of annealing the samples at higher ageing temperature showed that from 200 °C the hardness dropped to approx. 52 HV0.2 (Figure 17).

The tests performed revealed that DMLS elements have a slightly higher hardness than castings. This is due to the strong fragmentation of precipitates and phases rich in silicon or magnesium. During the heat treatment, the changes in mechanical properties are similar to those for a cast alloy, which indicates that it is not necessary to use different heat treatment conditions due to the manufacturing method. With long ageing times, both for castings and DLMS elements, the mechanical properties should be the same, providing the product with low porosity and fine structure.

## 4. Conclusions

The metallographic tests performed showed:

1. A favorable alloy structure after DMLS process with evenly distributed precipitates against the background of a solid aluminum solution. In the cast, the secretions were located mainly in the interdendritic areas, which caused the occurrence of areas prone to cracks.
2. In DMLS samples, in contrast to cast samples, no lamellar precipitates of silicon were observed, which indicates their better resistance to cracking.
3. The total proportion of porosity did not exceed 2.38% in the cast samples and was less than 0.03% in the DMLS samples.
4. Both printed and cast samples showed similar mechanical properties (hardness) achieved after long ageing time, i.e., 16 h at 170 °C. The maximum hardness was observed for the time of 8 h. In order to shorten the ageing time, the temperature can be slightly increased to approx. 180 °C.
5. At temperatures of 200 °C and higher ageing occured, which caused a significant reduction in hardness.

## Figures and Tables

**Table 1:** DMLS process parameters.

Scan speed (mm/s)	1300
Yb-fiber laser power (W)	310
Layer thickness (μm)	30
Protective atmosphere	Argon
Platform temperature (°C)	200

## Data Availability

The data presented in this study are available on request from the corresponding author.
